# External Control Augmentation Increases Estimates Precision for Finerenone plus Sodium-Glucose Cotransporter-2 Inhibitors

**DOI:** 10.1016/j.ekir.2026.106409

**Published:** 2026-03-04

**Authors:** Sascha van Boemmel-Wegmann, Chris Bauer, Alexandra Zerck, Alexander Hartenstein, Johannes Schuchhardt, Rachel Knapp, Robert Edfors, Alfredo E. Farjat, Michael Walsh

**Affiliations:** 1 Real World Evidence Center of Excellence, Global Medical & Evidence, Bayer AG, Berlin, Germany; 2 MicroDiscovery GmbH, Berlin, Germany; 3 Division of Cardiovascular Medicine, Department of Clinical Sciences, Danderyd Hospital, Karolinska Institute, Stockholm, Sweden; 4 Bayer BV, Clinical Statistics and Analytics, Hoofddorp, Netherlands; 5 Division of Nephrology, Department of Medicine, McMaster University, Hamilton, Ontario, Canada; 6 Department of Health Research Methods, Evidence and Impact, McMaster University, Hamilton, Canada; 7 Population Health Research Institute, Hamilton Health Sciences, McMaster University, Hamilton, Canada

**Keywords:** chronic renal insufficiency, diabetes mellitus type 2, electronic health records, mineralocorticoid receptor antagonists, sodium-glucose transporter 2 inhibitors

## Abstract

**Introduction:**

The FIDELITY pooled analysis of the FIDELIO-DKD and FIGARO-DKD trials showed a complementary benefit of finerenone and sodium-glucose cotransporter-2 inhibitors (SGLT-2is) in patients with chronic kidney disease (CKD) and type 2 diabetes mellitus (T2D). This study used US electronic health record (EHR) data to create an external control arm (ECA) to augment the FIDELITY comparator group and improve the precision of finerenone's treatment effect estimates.

**Methods:**

We identified eligible patients from EHR data who met the adapted FIDELITY criteria. These ECA patients were matched (1:1) to the pool of SGLT-2i users within the FIDELITY population using a linear programming method based on baseline demographics and clinical characteristics. We then calculated the treatment effects of finerenone on cardiovascular (CV) and kidney composite end points, hospitalization for heart failure (HHF), and all-cause mortality, incorporating the augmented ECA data.

**Results:**

Eligible ECA patients (*n* = 877) were matched to FIDELITY SGLT-2i users (*n* = 877), yielding a median (Q1–Q3) absolute standardized mean difference (ASMD) of 0.000 (0.000–0.004). ECA augmentation improved the precision of the trial estimates; data resembled the original FIDELITY estimates but with narrower 95% confidence interval (CI) ranges. For the CV end point and HHF, a significant benefit for finerenone + the SGLT-2i subgroup versus the augmented SGLT-2i controls was observed.

**Conclusion:**

Our findings demonstrate that an ECA can effectively augment underrepresented study populations in cardiorenal trials, enhancing the statistical precision of treatment effect estimates when there is sufficient homogeneity between the internal and external control groups.

## Graphical abstract

**Figure:**
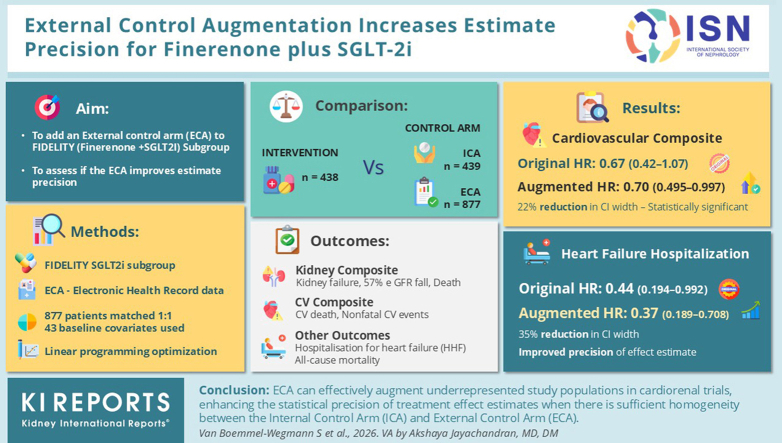

Subgroup analyses from clinical trials are typically not powered to gain reliable statistical inferences through hypothesis testing.^1^ In randomized clinical trials (RCTs) designed to detect a treatment effect across an overall population, treatment-effect-by-subgroup interactions are often underpowered and do not achieve statistical significance. However, given the lack of power, the absence of statistical significance does not imply the absence of treatment effect heterogeneity across subgroups.^1^

An ECA is a type of control group in which the data for the control participants are collected outside of the current RCT, such as from EHRs, medical claims databases, registries or historic (i.e., concluded) RCTs, to serve as a comparator for a treated group.^2^^,^^3^ The growing availability of high-quality real-world data (RWD) has led to increased endorsement of using these data sources to support clinical decision-making, with regulatory bodies in several countries and regions implementing frameworks supporting study designs that use both RCT and RWD.^3^ ECAs have been used in studies of diseases with high and predictable mortality and when comparison with randomized control arms would be unethical or infeasible (e.g., limited patient population sizes for rare diseases, single-arm oncology trials, or otherwise underrepresented patients in clinical trials).^4^^,^^5^ The US Food and Drug Administration suggests that patient-level RWD generating ECAs require careful attention to study design choices to minimize the threat of selection bias, confounding, information bias, and other aspects that may impact the validity of findings.^3^^,^^6^

FIDELITY, a prespecified pooled analysis of FIDELIO-DKD (NCT02540993) and FIGARO-DKD (NCT02545049), showed an independent and additive clinical effect of the concomitant use of finerenone and SGLT-2is in patients with T2D-associated CKD.^7^ In addition, this was reflected in the FIDELITY subgroup analysis reported by Rossing *et al.*^7^ The results showed wide CIs for the treatment effect estimates of comparative efficacy between patients who received SGLT-2is and patients who did not,^7^ indicating an inadequate sample size to provide precise estimates. SGLT-2is became a guideline-recommended treatment option for the management of CKD in patients with T2D during the clinical trial periods.^8^, ^9^, ^10^, ^11^ However, the uptake of SGLT-2is in patients with T2D-associated CKD has been slow in clinical practice, with 4.6% to 11.0% of patients receiving an SGLT-2i in 2019 and 2020.^12^ Furthermore, patients receiving SGLT-2is still progress toward kidney failure and CV events.^13^ Preclinical research of finerenone use in combination with an SGLT-2i (empagliflozin) has shown additive cardiorenal and survival benefits.^14^ A recently conducted RCT by Agarwal *et al.* confirmed a significantly greater reduction of urine albumin-to-creatinine ratio (UACR) in participants treated with finerenone plus empagliflozin, compared with those treated with either treatment alone.^15^ These results, along with recent RWD,^16^ suggest that the efficacy of combination therapy may exceed that of the respective monotherapies.

It was hypothesized that using an ECA to augment the internal control arm (ICA) of the FIDELITY SGLT-2i subgroup would increase the statistical power of treatment comparisons and consequently enhance the precision of effect estimates. The current study aimed to assess the feasibility of applying an ECA in pivotal cardiorenal trials to complement the indicative findings of the FIDELITY SGLT-2i–user subgroup analysis by augmenting the ICA using EHRs from a large US database.

## Methods

### Study Design and Objectives

This study consisted of a hybrid analysis of retrospective observational health care data from Optum EHR data matched to pooled trial data from FIDELITY. The objective of this study was to assess the feasibility of building an ECA of patients with T2D-associated CKD who use SGLT-2is in real-world clinical practice in order to augment the FIDELITY SGLT-2i subgroup analysis and determine whether this could be used to inform on the complementary use of finerenone with an SGLT-2i. The trial registration number was NCT05640180.

### Setting

The settings of the FIDELIO-DKD and FIGARO-DKD trials and the FIDELITY prespecified pooled analysis have been described previously.^10^^,^^11^^,^^17^ In addition to the FIDELITY data, the present study used Optum EHR data collected from January 1, 2013 to September 30, 2021 and includes longitudinal patient-level data from the US.^18^ The database was selected for this study following a formal feasibility and fit-for-purpose data assessment. Key factors included the large sample size of patients with T2D-associated CKD (anticipated *n* > 10,000), the availability of clinical laboratory data (e.g., estimated glomerular filtration rate [eGFR] and UACR) necessary to emulate trial eligibility criteria, and its wide-reaching representation of US clinical practice across insured and uninsured populations.

### Statistical Power Considerations

To assess the feasibility of ECA augmentation, power analyses and sample size calculations were performed based on the CV composite event rates and effect sizes observed in the reference RCT subgroup.^7^ Assuming a 5% significance level, the baseline power of the RCT-only subgroup (*n* = 877, 1:1 ratio) was approximately 60% for the observed CV composite hazard ratio (HR) of 0.67. Our calculations demonstrated that augmenting the control pool to achieve a 1:3 ratio (treatment: 438; augmented control: 1314) would increase the power to approximately 80% for an HR of 0.65 (Supplementary Figure S1). This design provided the necessary statistical precision to evaluate the treatment effect in this specific clinical subgroup.

### Cohort Selection

**Figure 1:**
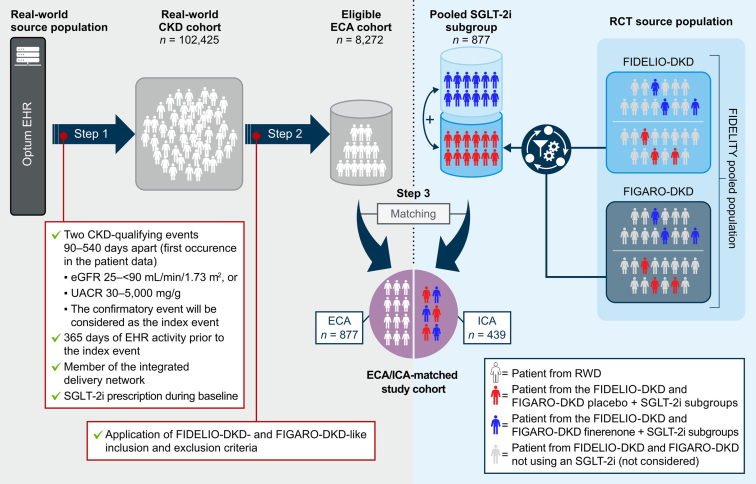
Selection process of an ECA cohort closely resembling the patients included in the FIDELITY analysis. Step 1: a set of universal selection criteria was applied for the identification of patients with CKD. The assignment of CKD stages in this study and the creation of the RW cohort based on inclusion and exclusion criteria are outlined in the following sections. Step 2: the “eligible ECA cohort” was created based on the application of translated inclusion and exclusion criteria from FIDELITY. In summary, the ECA cohort was comprised of adult patients with type 2 diabetes–associated CKD who qualified for finerenone therapy. Step 3: patients in the ICA were matched to those in the ECA to reduce confounding effects. CKD, chronic kidney disease; ECA, external control arm; eGFR, estimated glomerular filtration rate; EHR, electronic health record; ICA, internal control arm; RCT, randomized controlled trial; RW, real-world; RWD, real-world data; SGLT-2i, sodium-glucose co-transporter-2 inhibitor; UACR, urine albumin-to-creatinine ratio.

Eligibility criteria from FIDELIO-DKD and FIGARO-DKD were adapted to identify patients in the Optum EHR database similar to those from the FIDELITY pooled analysis (Figure 1). First, key inclusion criteria for the real-world CKD cohort were applied. Patients were required to have ≥ 2 CKD-qualifying laboratory values (an eGFR ≥ 25 ml/min per 1.73 m^2^ and < 90 ml/min per 1.73 m^2^, or UACR ≥ 30 mg/g and ≤ 5000 mg/g) 90 to 540 days apart to ensure disease chronicity. The date of a confirmatory measurement was considered as the index. In addition, patients were required to have ≥ 365 days of preindex EHR activity (baseline period) with ≥1 instances of documented care in the database during the 365-day baseline period, and ≥1 baseline prescriptions for an SGLT-2i. In a second selection step, further criteria were applied to generate an ECA-eligible cohort from the real-world CKD cohort. Selection criteria and their assessment periods (e.g., similar to the FIDELITY run-in period and screening visits) were aligned as closely as possible to FIDELIO-DKD and FIGARO-DKD (Supplementary Table S1). To ensure alignment with the RCT criteria, we applied a systematic feasibility assessment modeled after the process established by the RCT DUPLICATE initiative.^19^ Each FIDELITY selection criterion and outcome was evaluated for its “translatability” into the RWD setting using a “traffic light” logic: green denoted high conceptual overlap with laboratory values or validated algorithms, yellow indicated operable translation but with manageable limitations or the use of proxies, and red indicated criteria that were infeasible to replicate accurately and were thus only applied with limitations or excluded. The results of this assessment, confirming the viability of the emulation, are summarized in Supplementary Table S1. Patients were included if they were diagnosed with T2D at baseline,^20^ and were prescribed either angiotensin-converting enzyme inhibitors or angiotensin receptor blockers in a preindex period of 124 days. Key exclusion criteria included the following: 1 inpatient or 2 outpatient diagnoses of stroke, transient ischemic attack, acute coronary syndrome, or HHF (in a preindex period of 44 days); high serum potassium (> 4.8 mmol/l), glycated hemoglobin (> 12%), or UACR (> 5000 mg/g) in a preindex period of 124 days; hypertensive crisis, systolic heart failure, or left ventricular ejection fraction ≤ 40 % (in preindex period of 124 days); dialysis (in a preindex period of 208 days); and prescription of steroidal mineralocorticoid receptor antagonists or cytochrome P450 3A4 inhibitors or inducers within the baseline period. The eligible ECA cohort was further restricted to patients indexed between 2014 and 2019 to allow for sufficient follow-up of patients and to harmonize the RWD period with the FIDELITY analysis; data before 2014 and after 2019 were solely used for baseline assessment and follow-up, respectively.

### Patient Matching

Patients from the eligible ECA cohort were matched to patients receiving an SGLT-2i at baseline from FIDELITY on 43 medically informed baseline covariates using a direct optimization technique based on linear programming (Supplementary Table S2).^21^ This method was selected following a feasibility assessment comparing its performance against propensity score matching, genetic algorithm matching, and inverse odds weighting (Supplementary Figure S2). Although direct optimization requires the explicit definition of an objective function, in this study, the minimization of the ASMDs, and covariate-specific constraints, it was chosen because it maximizes the effective sample size while offering greater flexibility in balancing multiple disparate covariates compared with propensity score–based methods, which may overlook fine imbalances by collapsing covariates into a single score.^22^^,^^23^

### Outcomes

Patients within the ECA included in the study were followed-up with from day 1 after index until censorship (e.g., death, end of data availability, or until key trial entry criteria were violated [i.e., patient received a prescription for finerenone, 1 inpatient or 2 outpatient diagnoses of human immunodeficiency virus or cancer, a pregnancy diagnosis, a procedure code for bariatric surgery, receipt of a steroidal mineralocorticoid receptor antagonist {eplerenone or spironolactone}, or admission to a nursing home]). All analyses were conducted following the intention-to-treat principle relating to a patient’s SGLT2i use.

Study outcomes were chosen in alignment with the previous FIDELITY SGLT-2i subanalysis as follows: a kidney composite end point (time to the first occurrence of onset of kidney failure, a sustained decrease of eGFR ≥ 57% from baseline, or renal death), a CV composite end point (time to the first occurrence of CV death or nonfatal CV event without the occurrence of a death record within 45 days from the CV event), HHF, and all-cause mortality.^7^ The RCT end points were translated into RWD definitions using a combination of diagnosis and medical procedure codes and laboratory variables, as outlined in Supplementary Table S3, and their face validity assessed using the “traffic light” logic given the depicted limitations.

### Statistical Analysis

Matching was applied using a direct optimization technique based on linear programming, in which covariate imbalances were minimized. This approach directly targets covariate balance without the need for an intermediary propensity score,^21^ thereby providing greater flexibility to achieve a well-balanced cohort regardless of its underlying functional form. PyBalance was used to match patients from the eligible ECA cohort to patients in FIDELITY.^21^ The entire pooled FIDELITY subgroup of SGLT-2i users (*n* = 877), including patients from the finerenone and placebo arms, was used to find the most suitable matches from the eligible ECA cohort (matched 1:1). This resulted in a 1:2 ratio of ICA to ECA (439 vs. 877) and consequently, after augmenting the control arm, in a 1:3 ratio of active treatment (finerenone + SGLT-2i) versus SGLT-2i controls (ICA + ECA) (438 vs. 1316). The ASMD was plotted to visually assess the balance of variables, which consisted of baseline covariates used for the matching between the eligible ECA cohort and patients in FIDELITY. Although there is a lack of consensus about what value of ASMD represents meaningful residual imbalance in baseline covariates, some researchers have proposed 0.1 as an overall threshold.^24^ The empirical cumulative distribution function for continuous variables used for the matching was plotted and the area between the curves was used as a measure of distance. Side-by-side bar plots were used to display the frequency of binary variables used for matching.

To ensure the fitness-for-use of the EHR-derived end points, we conducted a prespecified feasibility assessment including heterogeneity tests. Specifically, these tests were performed to further assess comparability of clinical outcomes of the matched ECA cohort and ICA. Differences in incidence rates (IRs) between patients in the ICA and ECA were estimated using Poisson regression and tested using Wald tests with robust standard errors. HRs between the ICA and ECA were estimated using Cox proportional hazards models and tested using Wald tests with robust standard errors.

Baseline characteristics were summarized using descriptive statistics. Continuous variables were described using mean, SD, minimum, and quartiles. Categorical variables were reported as frequency counts and percentages. Incidence proportions and IRs with associated 95% CIs of the primary outcomes as reported by Rossing *et al.*^7^ were estimated over the entire course of follow-up. HRs and corresponding 95% CIs for treatment effect were calculated in the subset of SGLT-2i users at baseline from FIDELITY (finerenone + SGLT-2i vs. placebo + SGLT-2i) using stratified Cox models with the stratification factors used in FIDELITY.^7^ HRs and corresponding 95% CIs of the ECA-augmented subset of SGLT-2i users from FIDELITY were estimated using Cox proportional hazard models. Robust standard errors were calculated for the estimated Cox regression coefficients. In alignment with the reference analysis by Rossing *et al.*,^7^ we did not apply a formal adjustment for multiplicity. All end points were prespecified to be similar to the clinical trial definitions. Consequently, all reported *P*-values should be interpreted as nominal, and the focus should remain on the magnitude and direction of the treatment effect estimates (i.e., HRs) and their 95% CIs rather than strict hypothesis testing.

All analyses were conducted following the intention-to-treat principle. For the ECA, follow-up commenced on the day after the index date and continued until the occurrence of a study outcome, death, or end of data availability. To maintain the integrity of the treatment comparison, patients were censored if they initiated finerenone or other mineralocorticoid receptor antagonists (e.g., spironolactone, eplerenone) during the follow-up period, consistent with the censoring criteria used in the FIDELITY trial program.

Statistical analyses were performed in R, version 4.3.3 (http://www.r-project.org). The R package survival (v.3.5) was used for fitting the Cox proportional hazard models. The glm function from the R stats package was used to fit the Poisson regression. R packages lmtest (v.0.9) and sandwich (v.3.0) were used for the Wald test with robust standard errors.

## Results

### Creation of an ECA Through Matching

**Figure 2:**
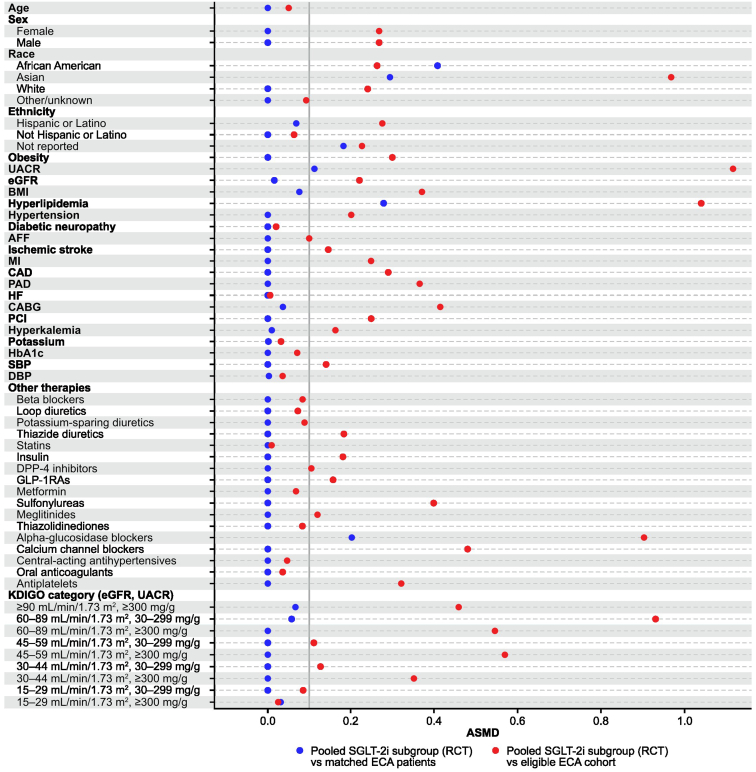
Absolute standardized mean difference before and after matching for the ECA cohorts (with matches for all patients in the ICA). A vertical line at 0.1 (10 %) is shown as a proposed threshold for balance of covariates.^24^ AFF, atrial fibrillation or flutter; ASMD, absolute standardized mean difference; BMI, body mass index; CABG, coronary artery bypass graft; CAD, coronary artery disease; DBP, diastolic blood pressure; DPP-4, dipeptidyl peptidase-4; ECA, external control arm; eGFR, estimated glomerular filtration rate; EHR, electronic health record; GLP-1RA, glucagon-like peptide-1 receptor agonist; HbA1c, glycated hemoglobin; HF, heart failure; ICA, internal control arm; KDIGO, Kidney Disease: Improving Global Outcomes; MI, myocardial infarction; PAD, peripheral artery disease; PCI, percutaneous coronary intervention; RCT, randomized controlled trial; SBP, systolic blood pressure; SGLT-2i, sodium-glucose co-transporter-2 inhibitor; UACR, urine albumin-to-creatinine ratio.

In total, 8272 patients were identified for the eligible ECA cohort. Baseline covariates for the matching method were selected from a diverse array of medically informed parameters covering demographic characteristics, comorbidities, comedications, and laboratory findings (Supplementary Table S2). Covariates with > 5% missing data or poor comparability between RWD and RCT data were not considered. Using linear programming with a set of 43 variables, 877 patients were successfully matched in a 1:1 ratio to the entire subset of SGLT-2i users from FIDELITY, resulting in a median (Q1–Q3) ASMD of 0.000 (0.000–0.004) (Figure 2, Supplementary Figures S2–S4).

Baseline characteristics of 439 pooled internal controls from FIDELITY were compared with 877 patients in the ECA. As indicated by the median ASMD, there was a high similarity between the baseline characteristics of ICA and ECA after matching (Table 1).

**Table 1:** Baseline characteristics in patients receiving an SGLT-2i at baseline, comparing the finerenone arm (from FIDELITY) versus the internal control arm (from FIDELITY) versus matched external control arm (from EHR)

Characteristics	Finerenone arm (from FIDELITY)	Internal control arm (from FIDELITY)^7^	Matched external control arm (from EHR)
*n*	438	439	877
Follow-up, yrs, median (Q1–Q3)	3.1 (2.4–3.9)	2.9 (2.3–3.8)	2.9 (1.5–4.1)
Age, yrs, mean ± SD	61.8 ± 9.7	61.6 ± 9.6	61.8 ± 10.7
Sex, *n* (%)			
Male	331 (75.6)	340 (77.4)	671 (76.5)
Female	107 (24.4)	99 (22.6)	206 (23.5)
Race, *n* (%)			
White	325 (74.2)	319 (72.7)	644 (73.4)
Asian	92 (21.0)	93 (21.2)	92 (10.5)
Black/African American	5 (1.1)	15 (3.4)	113 (12.9)
Other	16 (3.7)	12 (2.7)	28 (3.2)
Vital signs and laboratory measures			
SBP, mm Hg, mean ± SD	133.3 ± 14.8	133.4 ± 13.9	134.8 ± 12.7
HbA1c, %, mean ± SD	7.9 ± 1.2	8.0 ± 1.2	8.2 ± 1.3
Serum potassium, mmol/l, mean ± SD	4.3 ± 0.4	4.3 ± 0.4	4.3 ± 0.3
eGFR, ml/min per 1.73 m^2^, mean ± SD	66.8 ± 21.2	65.7 ± 21.0	66.6 ± 21.4
UACR, mg/g, median (Q1–Q3)	445.5 (186.0–959.2)	447.9 (186.1–930.7)	392.0 (135.0–825.9)
Medication use at baseline, *n* (%)			
RAS inhibitors	437 (99.8)	438 (99.8)	877 (100)
Beta-blockers	218 (49.8)	217 (49.4)	435 (49.6)
Loop diuretics	73 (16.7)	78 (17.8)	151 (17.2)
Thiazide diuretics	129 (29.5)	127 (28.9)	256 (29.2)
Statins	363 (82.9)	374 (85.2)	737 (84.0)
Potassium supplements	10 (2.3)	14 (3.2)	71 (8.1)
Potassium-lowering agents	5 (1.1)	2 (0.5)	0 (0.0)
Glucose-lowering therapies, *n* (%)			
Insulin and analogs	264 (60.3)	251 (57.2)	515 (58.7)
Metformin	352 (80.4)	340 (77.4)	692 (78.9)
Sulfonylureas	125 (28.5)	102 (23.2)	227 (25.9)
DPP-4 inhibitors	137 (31.3)	119 (27.1)	256 (29.2)
GLP-1RAs	86 (19.6)	81 (18.5)	167 (19.0)
Alpha-glucosidase inhibitors	25 (5.7)	10 (2.3)	8 (0.9)
Thiazolidinediones	34 (7.8)	25 (5.7)	59 (6.7)

DPP-4, dipeptidyl peptidase-4; eGFR, estimated glomerular filtration rate; EHR, electronic health record; GLP-1RA, glucagon-like peptide-1 receptor agonist; HbA1c, glycated hemoglobin; Q, quartile; RAS, renin–angiotensin system; RCT, randomized controlled trial; SBP, systolic blood pressure; SGLT-2i, sodium-glucose cotransporter-2 inhibitor; UACR, urine albumin-to-creatinine ratio.

**Figure 3:**
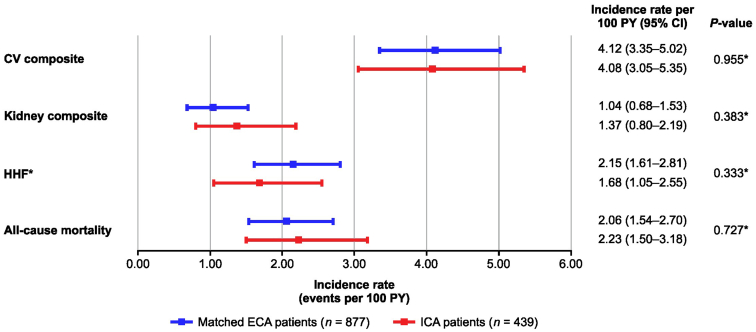
Incidence of outcomes in the ICA compared with the matched ECA. Comparison of patient characteristics in the ICA (FIDELITY) and the matched ECA. ∗Hypothesis test to compare incidence rates between the matched ECA vs. ICA controls. H_0_: Incidence rate for the ECA = Incidence rate for the ICA; H_a_: Incidence rate for the ECA ≠ Incidence rate for the ICA. CV, cardiovascular; ECA, external control arm; H_a_, alternative hypothesis; HHF, hospitalization for heart failure; H_0_, null hypothesis; ICA, internal control arm; PY, patient-years.

IRs of the 4 outcomes of interest exhibited a substantial overlap in their CIs between the ICA and ECA: CV composite IR/100 patient-years: 4.08 (95% CI: 3.05–5.35) versus 4.12 (95% CI: 3.35–5.02), kidney composite IR/100 patient-years: 1.37 (95% CI: 0.80–2.19) versus 1.04 (95% CI: 0.68–1.53), HHF IR/100 patient-years: 1.68 (95 %CI: 1.05–2.55) versus 2.15 (95% CI: 1.61–2.81), and all-cause mortality IR/100 patient-years: 2.23 (95% CI: 1.50–3.18) versus 2.06 (95% CI: 1.54–2.70) (Figure 3).

From the Wald tests with robust standard errors from Poisson and Cox regressions, we failed to reject the null hypothesis of equal IR and equal hazard rates, respectively. These results suggested sufficient data homogeneity between the ECA and ICA (Supplementary Tables S4 and S5, Figure 3). Based on these promising assessments, a decision was made to move ahead with the recalculation of effect estimated from FIDELITY using the RWD-enriched control group.

### Recalculation of Effect Estimates

**Figure 4:**
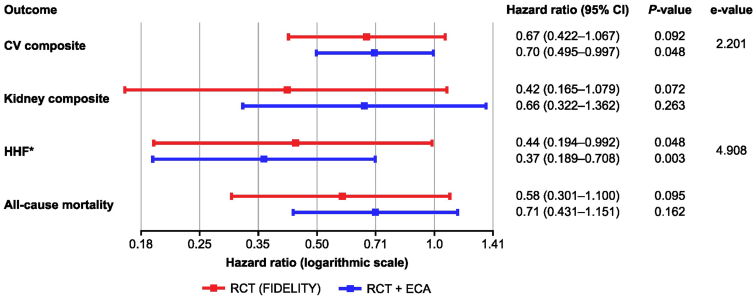
Effect estimates of the FIDELITY analysis (RCT) vs. the ECA-augmented RCT analysis (RCT + ECA). Comparison of the outcome effect estimates of the FIDELITY (RCT) data versus the ECA-augmented RCT data (RCT + ECA), including the corresponding e-values calculated for the end points yielding statistical significance. ∗Component of the CV composite. CI, confidence interval; CV, cardiovascular; ECA; external control arm; HHF, hospitalization for heart failure; PY, patient-years; RCT, randomized controlled trial.

Treatment effects were recalculated by combining the original pooled finerenone + SGLT-2i subgroup from FIDELITY and the ECA-augmented control arm; the resulting treatment-to-control group ratio was 1:3 (Figure 4). The range of 95% CIs was reduced for the CV composite end point, HHF, and all-cause mortality, representing an increase in the precision of previously reported effect estimates. Reductions were most prominent for the CV composite outcome (HR_augmented_: 0.70 [95% CI: 0.495–0.997; *P* = 0.048] vs. HR_original_: 0.67 [95% CI: 0.422–1.067; *P* = 0.092]) and HHF (HR_augmented_: 0.37 [95% CI: 0.189–0.708; *P* = 0.003] vs. HR_original_: 0.44 [95 % CI 0.194–0.992; *P* = 0.048]), with absolute 95% CI range reductions of 0.143 (22% precision gain) and 0.279 (35% precision gain), respectively (Supplementary Table S6). Although an increase in precision was not achieved for the kidney composite end point versus the FIDELITY analysis, the recalculated effect estimate indicated a nonsignificant treatment benefit for finerenone + SGLT-2i combination therapy similar to the nonaugmented results.

## Discussion

Our results highlight how the ECA methodology can enhance robust evidence generation in otherwise impractical clinical settings. As demonstrated through recalculating the FIDELITY treatment effect estimates, ECAs hold the potential to increase the precision of statistical analyses and to improve certainty in RCT inferences.^3^^,^^25^, ^26^, ^27^ This study evaluated the feasibility and performance of ECA methods beyond rare indications and in a broader cardiorenal population, where care standards evolve rapidly or certain groups may be underrepresented in pivotal clinical trials.^25^^,^^28^ We emphasize that the primary contribution of this work is methodological and the results should be viewed as confirmatory and complementary to the published clinical trial findings, not as providing fundamentally new clinical evidence.

The approach applied in this analysis is a recommended and straightforward way of using information from an external data source to enhance statistical power for the analysis of treatment effects. However, careful consideration of underlying assumptions is essential to ensure valid conclusions.^29^ Recognizing that the use of external controls may introduce statistical bias in effect estimates, applying appropriate methods and selecting suitable databases can help to manage type 1 error inflation while enhancing efficiency and power when suitable external controls are available.^30^

Beyond the method used in this analysis, other dynamic borrowing methods have been suggested, including Bayesian approaches such as power prior models (case weighted adaptive power prior model^30^), commensurate prior, meta-analytic predictive, robust meta-analytic predictive, and hierarchical models.^29^^,^^31^ Simulation studies by Damone *et al.* showed that, in specific scenarios, case weighted adaptive power prior achieved respectable power gains and well-controlled type 1 error across 5 different methods tested, closely followed by fixed weight power prior and propensity score matching methods.^30^ It is worth noting that no single adjustment method is universally superior to others; without randomization, no statistical adjustment method can guarantee that treatment assignment is independent of confounders. In some cases, the validity of different techniques relies on assumptions that cannot be fully verified from the data. A robust approach is to apply multiple statistical methods for covariate adjustment and assess the consistency of treatment effect estimates.^31^

The approach used in the current analysis provided encouraging results. Future research should compare the results of this analysis with those generated using dynamic borrowing methods, such as case weighted adaptive power prior. The augmented data generated by the current analysis support the data presented by Rossing *et al.*,^7^ which demonstrated the independent benefit of treatment with finerenone and SGLT-2is across several end points, such as the CV composite end point.

Our analysis should be contextualized in light of the recently published CONFIDENCE trial, which provided evidence on the effect of simultaneous initiation of finerenone and an SGLT-2i (empagliflozin) on albuminuria.^15^ The CONFIDENCE trial established that the combination therapy provides superior UACR reduction compared with monotherapy. In addition, recent actuarial analyses have used data from large-scale cardio-renal trials to project the significant long-term CV and kidney benefits of combining SGLT2 inhibitors and nonsteroidal mineralocorticoid receptor antagonists.^32^^,^^33^ The consistency of the favorable hard outcome treatment effect estimates observed in our ECA-augmented analysis, specifically the enhanced statistical certainty regarding the CV composite end point and HHF, is suggestive of a robust clinical benefit extending to CV outcomes in this patient subgroup. Thus, our ECA methodology provides supporting, complementary evidence, aligning with and strengthening the biological plausibility of the combination signals observed in the short-term UACR-focused CONFIDENCE randomized trial and lifetime gains in event-free and overall survival projected via actuarial analyses.

### Strengths and Limitations

Consideration must be given to the selection of the data source when planning to use an ECA approach to ensure harmonization of variables with those from the clinical trial and that appropriate, clinically relevant data are captured. Selection criteria and study outcomes from FIDELITY were carefully translated into the RWD setting, using validated algorithms, where available.^20^^,^^26^ Detailed face-validity assessments (Supplementary Table S1) were performed.

Although homogeneity was detected between the ECA and ICA, the ECA only comprised 877 patients, leading to a modest decrease in the range of the 95% CIs observed for the treatment effect estimates. Adding more patients to the ECA would theoretically increase the precision of these estimates; however, this increase would likely diminish beyond a certain point. An additional limitation was the geographic make-up of the ECA (derived from US clinical practice data), whereas FIDELITY data were derived from global prospective data collection. This difference constrains the external validity of the ECA findings regarding their direct application to non-US global populations without adjustment.^34^ For example, the racial distribution between FIDELITY and the ECA was inherently different and thus yielded higher ASMDs compared with other variables, indicating that matching was less favorable. This is represented by the proportional differences in racial identities of patients in the ICA and ECA (Table 1). Similarly, utilization of alpha-glucosidase inhibitors differed substantially between FIDELITY and the ECA. This is largely attributable to regional practice patterns, because alpha-glucosidase inhibitors are rarely prescribed in US clinical practice.^35^ Our subanalysis of FIDELITY by region showed very similar alpha-glucosidase inhibitor use in North American FIDELITY patients and the ECA (Supplementary Table S7). Furthermore, the original FIDELITY pooled analysis demonstrated that the treatment effect of finerenone was consistent across all geographic regions, with no significant interaction between region and treatment efficacy (e.g., CV composite HR: 0.84 [95% CI: 0.68–1.04] in North America vs. 0.86 [95% CI: 0.78–0.95] globally; *P*_interaction_ = 0.46).^17^ This alignment supports the transportability of our findings; it suggests that though the base populations differ, the treatment effects observed in US RWD can inform clinical expectations for similar patients in other jurisdictions when specific characteristics (like region-specific standard of care) are accounted for. By applying the linear programming approach, we were able to identify and match patients from RWD that were similar to FIDELITY patients across a majority of the covariates, although residual confounding of unmeasured characteristics cannot be ruled out. Furthermore, apparent imbalances in potassium supplement use (i.e., higher in the ECA) likely reflect differences in data capture definitions rather than actual use. The ECA used comprehensive National Drug Codes that capture any potassium-containing product, potentially including low-dose supplements or multivitamins, whereas clinical trial reporting typically focuses on prescription-strength supplementation for hypokalemia. Consequently, this difference should be interpreted as a measurement artifact rather than an indication of underlying clinical disparities between the patient populations of interest.

Although the direct optimization technique offers precise covariate balancing, we acknowledge that it requires explicit selection of an optimization function (i.e., minimizing ASMDs), which introduces analyst-driven choices regarding the matching structure.^23^ However, this direct control offers an advantage in this high-dimensional context. Unlike propensity score methods, where balance is an indirect byproduct of the estimated score, direct optimization allows for the simultaneous and transparent balancing of multiple individual confounders, reducing model dependence and the risk of misspecification. Our feasibility testing confirmed that direct optimization outperformed standard propensity score matching in reducing covariate imbalance (Supplementary Figure S2).

Furthermore, the definition of the index date in RWD required careful alignment with the RCT design to prevent immortal time bias. We defined the index date as the date of the second confirmatory laboratory measurement (verifying CKD chronicity), with follow-up commencing immediately thereafter. This ensured that no preindex time, that is, the interval between the initial and confirmatory laboratory values, was included in the outcome analysis.

We acknowledge that this approach introduces a degree of survival bias, because patients must survive the 90-to-540-day interval between qualifying laboratory values to enter the cohort. However, this design was necessary to strictly emulate the selection criteria of the FIDELIO-DKD and FIGARO-DKD trials, which required established CKD and stable background therapy before randomization. Effectively, the interval between the 2 real-world laboratory values serves as a proxy for the RCT screening and run-in periods, ensuring that the ECA population reflects the same established disease chronicity as the trial population.

One important limitation of this study is the inherent difference in outcome ascertainment between RCTs and RWD: end points in FIDELITY were thoroughly translated to RWD definitions, but differences may remain. Consequently, some degree of misclassification bias relative to the adjudicated trial end points cannot be ruled out. Laboratory values, such as eGFR or UACR, are collected at routine intervals in RCTs, whereas in clinical practice this happens irregularly and less frequently.^36^ Similarly, information bias may be introduced because patients with more severe symptoms may have more frequent hospital visits. Broader criteria were needed to capture multiple eGFR values, potentially compromising the specificity of distinguishing 2 acute eGFR decreases because of a chronic decline of kidney function. By using a complex algorithm for identifying composite kidney outcomes comprising components such as qualifying eGFR values, initiation of maintenance dialysis, kidney transplants, and renal death, we aimed to increase outcome specificity and thus decrease bias. Similar approaches were taken for other outcomes. Clinical outcomes from RCTs are typically adjudicated by a panel of experts, which aims to increase reliability through more accurate assessment of events. Despite events from the ECA of this study not being adjudicated, the incidence and effect estimates were similar. This is consistent with literature suggesting low value in adjudication especially of cardiorenal outcomes.^37^ By augmenting the ICA with external controls, differential misclassification of outcome events may have been introduced, which can bias results away from or towards the null. To mitigate this bias, we assessed homogeneity in cumulative IRs between the randomized ICA and the ECA for all outcomes before augmentation. Furthermore, sensitivity analyses (e.g., excluding death from composite end points) confirmed the consistency of these rates. This empirical validation suggests that, despite the methodological differences in definition, the EHR-derived outcomes served as valid proxies for the adjudicated events in this analysis. Nevertheless, some end points in both FIDELITY and the ECA (e.g., kidney-focused end points) had a low number of observed events, which may partially explain the persistently wide CIs for estimated treatment effects.

In addition, this study did not examine tolerability or adverse events related to combination treatment. It should be noted that SGLT-2i dose and type were not reported, limiting the ability to evaluate whether those factors modified reported outcomes. Our feasibility analyses showed that the quality of subsequent prescription information in the EHR data only allowed for the intent-to-treat approach.

Regarding residual confounding, our findings were assessed using quantitative bias analysis by calculating the e-value for the outcomes yielding a statistically significant difference between the finerenone arm and augmented controls (i.e., HHF, CV composite). An e-value of 4.9 for HHF (Figure 4) suggested that an unmeasured confounder would need to have a strong association with both the treatment and outcome to fully explain away the observed effect. For the CV composite, an e-value of 2.2 (Figure 4) indicated that an unmeasured confounder with a relatively moderate strength of association would be required to negate the observed effect. Hence, these results should be interpreted with caution and thus may not add further conclusions on top of the results published by Rossing *et al.*^7^

## Conclusion

Overall, these findings indicate that constructing an ECA from EHR RWD for augmentation of cardiorenal trial data is feasible. Through enrichment of the ICA-control group using an RWD-based ECA approach, this study increased the statistical precision of treatment comparisons previously generated within the FIDELITY analysis. This demonstrates the methodological value of ECAs in providing certainty for treatment effects in underrepresented clinical trial subgroups. Our results provide complementary support for combined use of finerenone + SGLT-2is in patients with CKD and T2D, indicating a statistically significant benefit for the CV composite end point.

## Disclosure

SvB-W was an employee at Bayer AG at the time of analysis and is currently an employee at Flatiron Health GmbH. CB was an employee at MicroDiscovery GmbH at the time of analysis; MicroDiscovery GmbH was paid by Bayer for statistical analyses. AZ was an employee at MicroDiscovery GmbH at the time of analysis; MicroDiscovery GmbH was paid by Bayer for statistical analyses. AH was an employee at Bayer AG. JS was an employee at MicroDiscovery GmbH at the time of analysis; MicroDiscovery GmbH was paid by Bayer for statistical analyses; JS is currently an employee at AlphaDiscovery GmbH. RK was an employee at Bayer AG. RE was an employee at Bayer AG at the time of analysis; currently an employee at Kancera AB. AEF was an employee at Bayer BV. MW reported receiving grants from the British Heart Foundation, Canadian Institutes of Health Research, Health Research Council (New Zealand), Medical Research Future Fund, National Health and Medical Research Council (Aus), Hamilton Academic Health Sciences Organization (HAHSO), and Vifor; consultancies from Alexion, Bayer, GSK, Otsuka, and Visterra; steering committees for studies funded by Bayer, Canadian Institutes of Health Research, Medical Research Future Fund, National Health and Medical Research Council (Aus), and Otsuka; data safety monitoring boards for studies funded by Hansa Pharmaceuticals, Medical Research Council (UK), National Institute of Health Research (UK), and Roche; event adjudication committees for studies funded by Dutch Kidney Foundation, Novo Nordisk; and employment with the Ontario Renal Network of Ontario Health.

## Supplementary Material

Supplementary File (PDF) **Figure S1.** Power as functions of the overall sample size. **Figure S2.** Standardized mean differences of ECA cohorts. **Figure S3.** Empirical cumulative distribution for continuous variables used for matching. **Figure S4.** Variable frequency for ECA cohorts. **Table S1.** Main FIDELIO-DKD and FIGARO-DKD inclusion and exclusion criteria that were applied to the real-world CKD cohort. **Table S2.** Baseline covariates used for matching. **Table S3.** Definition of outcomes. **Table S4.** IRs and Wald test *P*-values for the ICA and the matched ECA cohorts. **Table S5.** HRs and Wald test *P*-values for the ICA and the matched ECA cohorts. **Table S6.** CI widths of study outcomes for the analyses using the ICA versus ICA + ECA cohorts as control groups. **Table S7.** Baseline characteristics in patients receiving an SGLT-2i at baseline, comparing FIDELITY patients (region = North America) vs matched external controls (EHR). **STROBE Statement.** Checklist of items that should be included in reports of observational studies.

## Footnotes

Supplementary File (PDF)
**Figure S1.** Power as functions of the overall sample size. **Figure S2.** Standardized mean differences of ECA cohorts. **Figure S3.** Empirical cumulative distribution for continuous variables used for matching. **Figure S4.** Variable frequency for ECA cohorts. **Table S1.** Main FIDELIO-DKD and FIGARO-DKD inclusion and exclusion criteria that were applied to the real-world CKD cohort. **Table S2.** Baseline covariates used for matching. **Table S3.** Definition of outcomes. **Table S4.** IRs and Wald test *P*-values for the ICA and the matched ECA cohorts. **Table S5.** HRs and Wald test *P*-values for the ICA and the matched ECA cohorts. **Table S6.** CI widths of study outcomes for the analyses using the ICA versus ICA + ECA cohorts as control groups. **Table S7.** Baseline characteristics in patients receiving an SGLT-2i at baseline, comparing FIDELITY patients (region = North America) vs matched external controls (EHR).
**STROBE Statement.**
Checklist of items that should be included in reports of observational studies.
