# Supplementary material for: External Control Augmentation Increases Estimates Precision for Finerenone plus Sodium-Glucose Cotransporter-2 Inhibitors
Source: Kidney Int Rep. 2026 Mar 4;11(5):106409. doi: 10.1016/j.ekir.2026.106409 (PMC13088957; doi:10.1016/j.ekir.2026.106409)
Supplement: Supplementary File (PDF) — Figure S1. Power as functions of the overall sample size. Figure S2. Standardized mean differences of ECA cohorts. Figure S3. Empirical cumulative distribution for continuous variables used for matching. Figure S4. Variable frequency for ECA cohorts. Table S1. Main FIDELIO-DKD and FIGARO-DKD inclusion and exclusion criteria that were applied to the real-world CKD cohort. Table S2. Baseline covariates used for matching. Table S3. Definition of outcomes. Table S4. IRs and Wald test P-values for the ICA and the matched ECA cohorts. Table S5. HRs and Wald test P-values for the ICA and the matched ECA cohorts. Table S6. CI widths of study outcomes for the analyses using the ICA versus ICA + ECA cohorts as control groups. Table S7. Baseline characteristics in patients receiving an SGLT-2i at baseline, comparing FIDELITY patients (region = North America) vs matched external controls (EHR). STROBE Statement. Checklist of items that should be included in reports of observational studies. [file mmc1.pdf]

External Control Augmentation Increases Estimates Precision for Finerenone plus SGLT-2i

**Supplementary Table S.1.**	Main FIDELIO-DKD and FIGARO-DKD inclusion and exclusion criteria that were applied to the real-world CKD cohort.
**Supplementary Table S.2.**	Baseline covariates used for matching.
**Supplementary Table S.3.**	Definition of outcomes.
**Supplementary Table S.4.**	IRs and Wald test *P*-values for the ICA and the matched ECA cohorts.
**Supplementary Table S.5.**	HRs and Wald test *P*-values for the ICA and the matched ECA cohorts.
**Supplementary Table S.6.**	CI widths of study outcomes for the analyses using the ICA versus ICA + ECA cohorts as control groups.
**Supplementary Table S.7.**	Baseline characteristics in patients receiving an SGLT-2i at baseline, comparing FIDELITY patients (region = North America) vs matched external controls (EHR).
**Supplementary Figure S.1.**	Power as functions of the overall sample size.
**Supplementary Figure S.2.**	Standardized mean differences of ECA cohorts.
**Supplementary Figure S.3.**	Empirical cumulative distribution for continuous variables used for matching.
**Supplementary Figure S.4.**	Variable frequency for ECA cohorts.
**STROBE Statement**	Checklist of items that should be included in reports of observational studies

Supplementary tables

Supplementary Table S.1. Main FIDELIO-DKD and FIGARO-DKD inclusion and exclusion criteria that were applied to the real-world CKD cohort. (Right-hand column: green, high face-validity of the application in RWD; orange, application of criteria operable but with limitations; red, RWD translation of RCT criteria is potentially biased or infeasible).

RCT criteria	Trial	RWD translation for EHR data	Face-validity and potential limitations of the criteria application in RWD
		**Inclusion criteria**	
1) FIDELIO-DKD patients<br>Subjects with a clinical diagnosis of DKD based on either of the following criteria at the run-in and screening visits:<br>● Persistent **high albuminuria** defined as UACR of ≥ 30 mg/g (≥ 3.4 mg/mmol) but < 300 mg/g (< 33.9 mg/mmol) in 2 out of 3 first morning void samples and eGFR ≥ 25 but < 60 mL/min/1.73 m^2 (CKD-EPI) and presence of diabetic retinopathy in the medical history<br>OR<br>● Persistent **very high albuminuria** defined as UACR of ≥ 300 mg/g (≥ 33.9 mg/mmol) in 2 out of 3 first morning void samples and eGFR ≥ 25 but < 75 mL/min/1.73 m^2	FIDELIO-DKD	Diagnosis with CKD, for which either one of the following conditions must hold true:<br>● An index eGFR value of ≥ 25 and < 60 mL/min/1.73 m^2 and<br>○ An index UACR of ≥ 30 and < 300 mg/g, and<br>○ The presence of at least one ICD code for diabetic retinopathy on the medical records in any position prior to the index date; or<br>● An index eGFR value of ≥ 25 and < 75 mL/min/1.73 m^2, and an index UACR of ≥ 300 and < 5000 mg/g	Lab values of UACR are limited in EHR data. Even though multiple eGFR and UACR values are used for inclusion of a real-world patient, disease persistence cannot be ascertained as accurately as in the RCT. This approach may introduce misclassification bias of patients with “persistent” high albuminuria.
2) FIGARO-DKD patients	FIGARO-DKD	Diagnosis with CKD, for which either one of the following conditions must hold true:	Lab values of UACR are limited in EHR data. Even though multiple eGFR and UACR values are used for inclusion of a

Subjects with a clinical diagnosis of DKD based on either of the following criteria at the run-in and screening visits:<br>● Persistent **high albuminuria** defined as UACR of ≥ 30 mg/g (≥ 3.4 mg/mmol) but < 300 mg/g (< 33.9 mg/mmol) in 2 out of 3 first morning void samples and eGFR ≥ 25 but ≤ 90 mL/min/1.73 m^2<br>OR<br>● Persistent **very high albuminuria** defined as UACR of ≥ 300 mg/g (≥ 33.9 mg/mmol) in 2 out of 3 first morning void samples and eGFR ≥ 60 mL/min/1.73 m^2		● An index eGFR value of ≥ 25 and < 90 mL/min/1.73 m^2, and an index UACR of ≥30 and < 300 mg/g, or<br>● An index eGFR value of ≥ 60 mL/min/1.73 m^2, and index UACR value of ≥ 300 and < 5000 mg/g	real-world patient, disease persistence cannot be ascertained as accurately as in the RCT. This approach may introduce misclassification bias of patients with "persistent" high albuminuria
Men or women aged 18 years and older	FIDELIO-DKD, FIGARO-DKD	≥ 18-years-old on the index date as identified from the patient demographic information	
Women of childbearing potential can only be included in the study if a pregnancy test is negative at the screening visit and if they agree to use adequate contraception	FIDELIO-DKD, FIGARO-DKD	N/A<br>(for the exclusion of patients with active pregnancy, see below)	Pregnancy tests and contraception use is not adequately captured in EHR data
Diagnosed with T2D	FIDELIO-DKD, FIGARO-DKD	To identify patients with T2D in Optum EHRs, we used a phenotyping algorithm developed by Sun and Hernandez-Boussard (Stanford University, 2020), which extracts individuals with diabetes and classifies the diabetes sub-type from EHRs. The phenotype algorithm includes diagnoses during the baseline period	
Prior treatment with ACEis and ARBs as follows:<br>1. For at least 4 weeks prior to the run-in visit, subjects should be	FIDELIO-DKD, FIGARO-DKD	Patients treated with an ACEi or ARB, not both, during 124 days[a] prior to the index date, assessed by the presence of at least one prescription record, inpatient	Medication utilization in EHR data is derived from physician prescription, drug administration procedures, or patient-reported records. However, no

treated with either an ACEi or ARB, or both<br>2. Starting with the run-in visit, subjects should be treated with only an ACEi or ARB<br>3. For at least 4 weeks prior to the screening visit, subjects should be treated with the maximum tolerated labeled dose (but not below the minimal labeled dose) of only an ACEi or an ARB (not both) preferably without any adjustments to dose or choice of agent or to any other antihypertensive or antiglycemic treatment		administration, or patient-reported utilization with a respective NDC code	information is available as to whether the patient filled the prescription or took the drug, which can cause misclassification of users and nonusers. In addition, specifics on strength, days of supply, etc. of the prescriptions are limited and thus cannot be used
Serum potassium ≤ 4.8 mmol/L at both the run-in and the screening visits	FIDELIO-DKD, FIGARO-DKD	[see exclusion criteria below]	
SGLT-2i use at baseline:<br>● Canagliflozin<br>● Dapagliflozin<br>● Empagliflozin<br>● Ertugliflozin<br>● Ipragliflozin<br>● Luseogliflozin<br>● Tofogliflozin	FIDELIO-DKD, FIGARO-DKD	Patients treated with an SGLT-2i during 124 days[a] prior to the index date, assessed by the presence of at least one prescription record, inpatient administration, or patient-reported utilization with a respective NDC code for one of the following FDA approved compounds:<br>● Canagliflozin<br>● Dapagliflozin<br>● Empagliflozin<br>● Ertugliflozin<br>Other SGLT-2is are not FDA approved	Medication utilization in EHR data is derived from physician prescription, drug administration procedures, or patient-reported records. However, no information is available as to whether the patient filled the prescription or took the drug, which can cause misclassification of users and nonusers. In addition, specifics on strength, days of supply etc. of the prescriptions are limited and thus cannot be used

Exclusion criteria

**Medical and surgical history**			
[See inclusion criteria above] Serum potassium ≤ 4.8 mmol/L at both the run-in and screening visits]	FIDELIO-DKD, FIGARO-DKD	The occurrence of one or more laboratory value of serum potassium > 4.8 mmol/L or a diagnosis of hyperkalemia during 124 days[a] prior to the index date	
Known significant nondiabetic renal disease, including clinically relevant renal artery stenosis	FIDELIO-DKD, FIGARO-DKD	N/A	Any ICD codes used to diagnose significant renal disease can apply regardless of whether it is due to diabetes or not. Therefore, based on the ICD codes it would be unclear whether or not the renal disease is of nondiabetic origins
UACR > 5000 mg/g (> 565 mg/mmol) at the run-in or screening visit	FIDELIO-DKD, FIGARO-DKD	One or more readings of UACR > 5000 mg/g during 124 days[a] prior to the index date	This approach may introduce misclassification bias of patients due to the low number of UACR lab values in EHR data
HbA1c > 12 % (> 108 mmol/mol) at the run-in or screening visit	FIDELIO-DKD, FIGARO-DKD	At least one HbA1c lab value > 12% (> 108 mmol/mol) during 124 days[a] prior to the index date	
Uncontrolled arterial hypertension with mean sitting SBP ≥ 170 mmHg or mean sitting DBP ≥ 110 mmHg at the run-in visit or mean sitting SBP ≥ 160 mmHg or mean sitting DBP ≥ 100 mmHg at the screening visit	FIDELIO-DKD, FIGARO-DKD	The occurrence of ≥ 2 blood pressure measurements with a mean of SBP ≥ 170 mmHg or DBP ≥ 110 mmHg or one inpatient or two outpatient diagnoses[b] of a hypertensive crisis during 124 days[a] prior to the index date	BP measurement information is underreported in EHR data and tends to be captured more often in extreme cases (high increase or decrease). Patients with significantly increased values should be excluded; therefore, this systematic bias has limited influence on our study sample
Mean SBP < 90 mmHg at the run-in or screening visit	FIDELIO-DKD, FIGARO-DKD	The occurrence of ≥ 2 blood pressure measurements with a mean of SBP < 90 mmHg of all SBP values occurring in the patient record during 124 days[a] prior to the index date	BP measurement information is underreported in EHR data and tends to be captured more often in extreme cases (high increase or decrease). Patients with significantly decreased values should be excluded; therefore, this

			systematic bias has limited influence on our study sample
Subjects with a clinical diagnosis of chronic heart failure with reduced ejection fraction and persistent symptoms (New York Heart Association class II–IV) at the run-in visit (class 1A recommendation for mineralocorticoid receptor antagonists)	FIDELIO-DKD, FIGARO-DKD	At least one observed measure for LVEF ≤ 40% on the patient observation table or one inpatient or two outpatient diagnosis codes[b] for systolic HF during 124 days[a] prior to the index date	Ejection fraction information is underreported in EHR data and tends to be captured more often in extreme cases (high increase or decrease). Patients with significantly decreased values should be excluded; therefore, this systematic bias likely has limited influence on our study sample
Stroke, transient ischemic cerebral attack, acute coronary syndrome, or hospitalization for worsening heart failure, in the last 30 days prior to the screening visit	FIDELIO-DKD, FIGARO-DKD	At least one inpatient or two outpatient diagnoses of[b]:<br>● Stroke<br>● Transient ischemic cerebral attack<br>● Acute coronary syndrome<br>● HHF<br>44 days[c] prior to the index date	
Dialysis for acute renal failure within 12 weeks prior to the run-in visit	FIDELIO-DKD, FIGARO-DKD	At least one dialysis procedure defined by the respective diagnosis or procedure code or one inpatient or two outpatient diagnoses[b] of acute kidney failure on the patient medical record in any position within 208 days[c] prior to the index date	
Renal allograft in place or a scheduled kidney transplant within the next 12 months from the run-in visit	FIDELIO-DKD, FIGARO-DKD	Kidney transplantation as identified by the occurrence by either one inpatient or two outpatient diagnoses or a procedure code on the patient medical records in any position prior to the index date	All patient data prior to index date is used to identify kidney transplants. However, a planned procedure after the index date cannot be ascertained on index. Patients receiving a kidney transplant were censored on the day of the procedure
Addison's disease	FIDELIO-DKD, FIGARO-DKD	At least one inpatient or two outpatient diagnoses codes[b] for Addison's disease during baseline	

Confidential - Not for Public Consumption or Distribution

Hepatic insufficiency classified as Child-Pugh C	FIDELIO-DKD, FIGARO-DKD	A respective diagnosis code for significant liver disease must be found on the patient medical record in any position at least once in the baseline period, and ALT/AST > 2.5 × the ULN.<br>ALT upper limit was set to 33 units/L for males and 25 units/L for females; AST upper limit was set to 40 units/L for males and 32 units/L for females. Levels must have been found on the medical record in the 30-day period prior to the index date. The closest values to the index date were used	The Child-Pugh score is difficult to validly assess in EHR, as it requires that there are no missing data in various lab tests and entry of encephalopathy and ascites diagnosis codes within a reasonable amount of time. With missing data in RWD it is unlikely to have high specificity. Instead, a 'severe liver disease' proxy medical concept combining diagnosis codes and lab values was used
**Medication and drug use**			
Concomitant therapy with eplerenone, spironolactone, any renin inhibitor, or potassium-sparing diuretic that cannot be discontinued at least 4 weeks prior to the screening visit	FIDELIO-DKD, FIGARO-DKD	Prescription, inpatient administration, or patient reported use within 90 days[d] prior to the index date of:<br>● Eplerenone or spironolactone<br>● Any renin inhibitor<br>● Potassium-sparing diuretic	Medication utilization in EHR data is derived from physician prescription, drug administration procedures, or patient-reported records. However, no information is available as to whether the patient filled the prescription or took the drug, which can cause misclassification of users and nonusers. In addition, specifics on strength, days of supply, etc., of the prescriptions are limited and thus cannot be used
Concomitant therapy with both an ACEi and ARB that cannot be discontinued for the purpose of the study	FIDELIO-DKD, FIGARO-DKD	[Concomitant ACEi and ARB therapy already addressed previously]	
Concomitant therapy with potent CYP3A4 inhibitors or inducers (to be stopped at least 7 days before randomization)	FIDELIO-DKD, FIGARO-DKD	At least one prescription, inpatient administration or a self-reported medication record with the respective NDC code for potent CYP3A4 inhibitors or inducers during baseline	Medication utilization in EHR data is derived from physician prescription, drug administration procedures, or patient-reported records. However, no information is available as to whether the patient filled the prescription or took the drug, which can cause misclassification of users and nonusers. In addition,

			specifics on strength, days of supply etc. of the prescriptions are limited and thus cannot be used
**Other**			
Any other condition or therapy that would make the subject unsuitable for this study and will not allow participation for the full planned study period (e.g., active malignancy or other condition limiting life expectancy to less than 12 months)	FIDELIO-DKD, FIGARO-DKD	Patients with any type of primary or secondary malignant neoplasm, except nonmelanoma skin cancer, as defined by the occurrence of at least one inpatient or two outpatient diagnosis codes[b] during baseline. In addition, patients with a baseline prescription of finerenone were excluded from the study	
Pregnant or breast-feeding or intention to become pregnant during the study	FIDELIO-DKD, FIGARO-DKD	Active pregnancy or breast-feeding, defined by the occurrence of at least one inpatient or two outpatient diagnosis codes[b] during baseline	

[a]124 days was chosen to mirror the maximum duration of run-in period (4–16 weeks) + screening visits (2 weeks) in the RCTs.

[b]For outpatient diagnoses, the two diagnoses needed to occur on two different days not longer than 30 days apart, whereas the second diagnosis is the confirmatory event.

[c]Chosen to mirror the RCT criterion, considering run-in period (4–16 weeks) and/or screening visit (2 weeks), whichever applies.

[d]90 days chosen to capture chronically prescribed medications with a maximum allowed days supply of 3 months.

Abbreviations: ACEi, angiotensin-converting-enzyme inhibitor; ALT, alanine aminotransferase; ARB, angiotensin receptor blocker; AST, aspartate aminotransferase; BP, blood pressure; CKD, chronic kidney disease; CKD-EPI, Chronic Kidney Disease Epidemiology Collaboration; CYP3A4, cytochrome P450 3A4; DBP, diastolic blood pressure; DKD, diabetic kidney disease; eGFR, estimated glomerular filtration rate; EHR, electronic health record; FDA, US Food and Drug Administration; HbA1c, glycated hemoglobin; HF, heart failure; HHF, hospitalization for heart failure; ICD, International Classification of Diseases; LVEF, left ventricular ejection fraction; N/A, not applicable; NDC, National Drug Codes; RCT, randomized controlled trial; RWD, real-world data; SBP, systolic blood pressure; SGLT-2i, sodium-glucose co-transporter-2 inhibitor; T2D, type 2 diabetes; UACR, urine albumin-to-creatinine ratio; ULN, upper limit of normal.

Supplementary Table S.2. Baseline covariates used for matching.

**Demographic parameters**	Age (years), BMI, ethnicity, gender, index year category (index year prior to 2019 or after 2019), obesity, race
**Medical history parameters**	Atrial fibrillation or flutter, coronary artery bypass graft, coronary artery disease, diabetic neuropathy, heart failure, hyperkalemia, hyperlipidemia, hypertension, ischemic stroke, myocardial infarction, percutaneous coronary intervention, peripheral arterial disease
**Medication parameters**	Alpha-glucosidase blockers, beta blockers, calcium channel blockers, central acting antihypertensives, biguanides, loop diuretics, potassium sparing diuretics, thiazide diuretics, insulin, DPP-4 inhibitors, GLP-1RAs, sulfonylureas, meglitinides, thiazolidinediones, statins, antiplatelets, oral anticoagulants
**Lab parameters (considered only parameters with < 5% missing values)**	eGFR, UACR, KDIGO category, potassium, HbA1c, diastolic blood pressure, systolic blood pressure

Abbreviations: BMI, body mass index, DPP-4, dipeptidyl peptidase-4; eGFR, estimated glomerular filtration rate; GLP-1RA, glucagon-like peptide-1 receptor agonist; HbA1c, glycated hemoglobin; KDIGO, Kidney Disease: Improving Global Outcomes; UACR, urine albumin-to-creatinine ratio.

Supplementary Table S.3. Definition of outcomes.

RCT criteria	Criterion in which trial? (FIDELIO-DKD/ FIGARO-DKD)	RWD translation (Optum® EHR)	Face-validity and potential limitations of the criteria application in RWD
1) FIDELIO-DKD<br>Kidney composite endpoint:<br>The primary efficacy variable will be the time to the first occurrence of the composite endpoint of<br>● Onset of kidney failure,<br>● A sustained decrease of eGFR ≥40% from baseline over at least 4 weeks, or<br>● Renal death	FIDELIO-DKD, FIGARO-DKD (part of the secondary efficacy variables)	The primary efficacy variable will be the time to the first occurrence of the composite endpoint of:<br>● Onset of kidney failure, defined as<br>○ Two different eGFR test results < 15 mL/min/1.73 m^2 separated by at least 90 days; eGFR determinations should be based on only outpatient measurements (inpatient measurements are more likely to be associated with transient changes in eGFR associated with acute events)<br>○ The initiation of maintenance dialysis (i.e., PD or HD) in the outpatient setting, defined as follows:<br>1) The first occurrence of a medical code for PD in an outpatient setting, **OR**<br>2) The occurrence of ≥ 3 medical codes for HD in an outpatient setting on different days for at least 45 days, with no gaps longer than 21 days. The outcome will be set at the confirmatory event (i.e., the first medical code occurring between 45 and 90 days after the initial hemodialysis code)<br>Furthermore,<br>▪ If the previous case definition has not been satisfied for an occurring HD code during	In EHR data, cause of death can only be inferred via a timely connection of diagnoses, procedure codes or lab values around the date of death. In addition, date of death is only available as month and year. Therefore, we applied an algorithm (outlined below) to identify renal death in the EHR data. The performance of the "renal death" algorithm is not validated and therefore the PPV is unclear. To understand the extent of the influence of "renal death" on the incidence rates, we will run two sets of sensitivity analyses. Firstly, we excluded "renal death" from both the RCT and RWD criteria and compared incidence rates of the outcomes to the original definition. Secondly, we replaced the cause-specific death component with all-cause mortality in the composite outcome, re-analyze and compare the results to the primary analysis

		follow-up, take the next occurrence and re-apply the above criteria<br>▪ If a patient dies within the 45-day period after the occurrence of an outpatient dialysis procedure and thus chronicity of dialysis could not be ascertained, the date of death will be considered as the day of the ESKD event<br>▪ Kidney transplant, identified through procedure coding for kidney transplant procedures or a recorded diagnosis indicating kidney transplant<br>● A sustained decrease of eGFR ≥ 40% from baseline, or<br>● Renal death	
2) FIGARO-DKD<br>Cardiovascular composite endpoint:<br>The primary efficacy variable will be the time to first occurrence of the composite endpoint of:<br>● CV death, or<br>● Nonfatal CV event (i.e., myocardial infarction, stroke or HHF)	FIGARO-DKD, FIDELIO-DKD (part of the secondary efficacy variables)	The primary efficacy variable will be the time to first occurrence of the composite endpoint of:<br>● CV death<br>● Nonfatal CV event, defined as either<br>○ An inpatient diagnosis for myocardial infarction in the primary or discharge diagnosis position<br>○ An inpatient diagnosis for stroke in the primary or discharge diagnosis position, or<br>○ An inpatient diagnosis for HF in the primary or discharge diagnosis position<br>without the occurrence of a death record within 45 days from the CV event	In EHR data, cause of death can only be inferred via a timely connection of diagnoses, procedure codes or lab values around the date of death. In addition, date of death is only available as month and year. Therefore, we applied an algorithm (outlined below) to identify CV death in the EHR data. The performance of the "CV death" algorithm is not validated and therefore the PPV is unclear. To understand the extent of the influence of "CV death" on the incidence rates, we ran two sets of sensitivity analyses Firstly, we excluded "CV death" from both the RCT and RWD criteria and compare incidence rates of the outcomes to the original definition. Secondly, we replaced the cause-specific death component with all-cause mortality in the composite outcome, re-analyze and compared the results to the primary analysis

Time to all-cause mortality	FIDELIO-DKD, FIGARO-DKD	Time to all-cause mortality	Time to all-cause mortality in RWD may be biased because in EHR data, time of death is only available as month and year. We attempted to minimize the bias by assigning the 15th day of each month as the date of death
HHF	FIDELIO-DKD, FIGARO-DKD	An inpatient diagnosis for HF in the primary or discharge diagnosis position	Previous research has shown that this approach yields a positive predictive value of 84–100 % [31]

Abbreviations: CV, cardiovascular; eGFR, estimated glomerular filtration rate; EHR, electronic health record; ESKD, end-stage kidney disease; HD, chronic hemodialysis; HF, heart failure; HHF, hospitalization for heart failure; PD, peritoneal dialysis; PPV, positive-predictive value; RCT, randomized controlled trial; RWD, real-world data.

Supplementary Table S.4. IRs and Wald test *P*-values for the ICA and the matched ECA cohort.

Outcome	3 years			4 years			Total time span		
	ECA IR	ICA IR	*P*-value	ECA IR	ICA IR	*P*-value	ECA IR	ICA IR	*P*-value
**CV composite endpoint**	4.540	4.540	.999	4.300	4.200	.900	4.120	4.080	.955
**Kidney composite endpoint**	0.715	0.923	.535	0.882	1.240	.316	1.040	1.370	.383
**HHF**	2.440	1.770	.230	2.150	1.730	.405	2.150	1.680	.333
**All-cause mortality**	1.930	2.080	.777	1.970	2.070	.838	2.060	2.23	.727

Abbreviations: CV, cardiovascular; ECA, external control arm; HHF, hospitalization for heart failure; ICA, internal control arm; IR, incidence rate.

Confidential - Not for Public Consumption or Distribution

Supplementary Table S.5. HRs and Wald test *p*-values for the ICA and the matched ECA cohort.

Outcome	3 years		4 years		Total time span	
	**HR**	***P*-value**	**HR**	***P*-value**	**HR**	***P*-value**
**CV composite endpoint**	0.997 (0.703–1.413)	0.986	0.966 (0.688–1.356)	0.841	0.942 (0.672–1.320)	0.729
**Kidney composite endpoint**	1.290 (0.570–2.900)	0.544	1.500 (0.767–2.943)	0.236	1.570 (0.837–2.950)	0.160
**HHF**	0.726 (0.430–1.226)	0.231	0.790 (0.478–1.310)	0.361	0.751 (0.456–1.238)	0.261
**All-cause mortality**	1.070 (0.644–1.792)	0.784	1.050 (0.652–1.796)	0.836	1.150 (0.726–1.815)	0.556

Abbreviations: CV, cardiovascular; ECA, external control arm; HHF, hospitalization for heart failure; HR, hazard ratio; ICA, internal control arm.

Supplementary Table S.6. CI widths of study outcomes for the analyses using the ICA versus ICA + ECA cohorts as control groups. HRs & 95 % CIs from Rossing et al. (PMID: 35972218).

Outcome	Analysis Type	HR (95% CI)	CI Width	Width Reduction
**CV Composite**	ICA	0.67 (0.422–1.067)	0.645	--
	ICA + ECA	0.70 (0.495–0.997)	0.502	**- 0.143 (22% reduction)**
**Kidney Composite**	ICA	0.42 (0.165–1.079)	0.914	--
	ICA + ECA	0.66 (0.322–1.362)	1.040	**+ 0.126 (--)**
**HHF**	ICA	0.44 (0.194–0.992)	0.798	--
	ICA + ECA	0.37 (0.189–0.708)	0.519	**- 0.279 (35% reduction)**
**All-cause mortality**	ICA	0.58 (0.301-1.100)	0.799	--
	ICA + ECA	0.71 (0.431-1.151)	0.720	**- 0.079 (10% reduction)**

Abbreviations: CV, cardiovascular; CI, confidence interval; ECA, external control arm; HHF, hospitalization for heart failure; HR, hazard ratio; ICA, internal control arm.

Supplementary Table S.7. Baseline characteristics in patients receiving an SGLT-2i at baseline, comparing FIDELITY patients (region = North America) vs matched external controls (EHR).

	FIDELITY patients from North America (RCT)	External control arm (EHR)
**n (%)**	**148**	**877**
**Follow-up, years, median (Q1–Q3)**	3.4 (2.5–4.2)	2.9 (1.5–4.1)
**Age, years, mean ± SD**	62.8 ± 10.5	61.8 ± 10.7
**Male**	107 (72.3%)	671 (76.5%)
**Female**	41 (27.7%)	206 (23.5%)
**White**	117 (79.1%)	644 (73.4%)
**Asian**	12 (8.1%)	92 (10.5%)
**Black/African American**	17 (11.5%)	113 (12.9%)
**Other**	2 (1.4%)	28 (3.2%)
**SBP, mmHg, mean ± SD**	129.7 ± 15.3	134.8 ± 12.7
**HbA1c, %, mean ± SD**	7.9 ± 1.2	8.2 ± 1.3
**Serum potassium, mmol/L, mean ± SD**	4.3 ± 0.4	4.3 ± 0.3
**eGFR, mL/min/1.73 m^2,mean ± SD**	64.1 ± 20.0	66.6 ± 21.4
**UACR, mg/g, median (Q1–Q3)**	359.8 (97.4–830.7)	392.0 (135.0–825.9)
**RAS inhibitors**	147 (99.3%)	877 (100%)
**Beta-blockers**	81 (54.7%)	435 (49.6%)
**Loop diuretics**	31 (21.0%)	151 (17.2%)
**Thiazide diuretics**	47 (31.8%)	256 (29.2%)
**Statins**	133 (89.9%)	737 (84.0%)

Confidential - Not for Public Consumption or Distribution

**Potassium supplements**	7 (4.7%)	71 (8.1%)
**Potassium-lowering agents**	1 (0.7%)	0 (0%)
**Insulin and analogs**	100 (67.6%)	515 (58.7%)
**Metformin**	96 (64.9%)	692 (78.9%)
**Sulfonylureas**	41 (27.7)	227 (25.9%)
**DPP-4 inhibitors**	42 (28.4%)	256 (29.2%)
**GLP-1RAs**	34 (23.0%)	167 (19.0%)
**Alpha-glucosidase inhibitors**	1 (0.7%)	8 (0.9%)
**Thiazolidinediones**	8 (5.4%)	59 (6.7%)

Abbreviations: DPP-4, dipeptidyl peptidase-4; eGFR, estimated glomerular filtration rate; EHR, electronic health record; GLP-1RA, glucagon-like peptide-1 receptor agonist; HbA1c, glycated hemoglobin; Q, quartile; RAS, renin–angiotensin system; RCT, randomized controlled trial; SBP, systolic blood pressure; SD, standard deviation; SGLT-2i, sodium-glucose co-transporter-2 inhibitor; UACR, urine albumin-to-creatinine ratio.

Supplementary figures

Supplementary Figure S.1. Power as functions of the overall sample size.

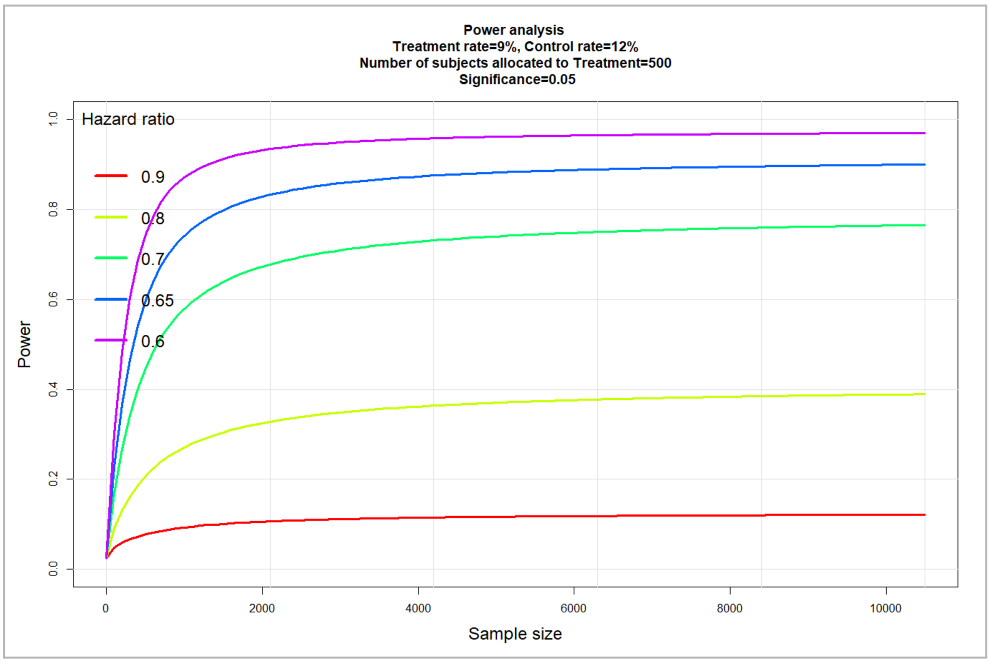

Assuming 500 patients are allocated to treatment, significance level 5% and the probabilities of observing an event during the maximum time period of the study (3 years) are 9% and 12% for treatment and control, respectively (roughly three times the estimated IRs for the CV composite). X-axis: Overall sample size. Y-axis: Statistical Power (0 to 1). Plotted Functions: Hazard ratios of 0.6, 0.65, 0.7, 0.8, and 0.9. Key Highlight: The point representing the 1:3 ratio (N=1,752 total) showing ~80% power for HR=0.65.

Supplementary Figure S.2. Standardized mean differences of ECA cohorts.

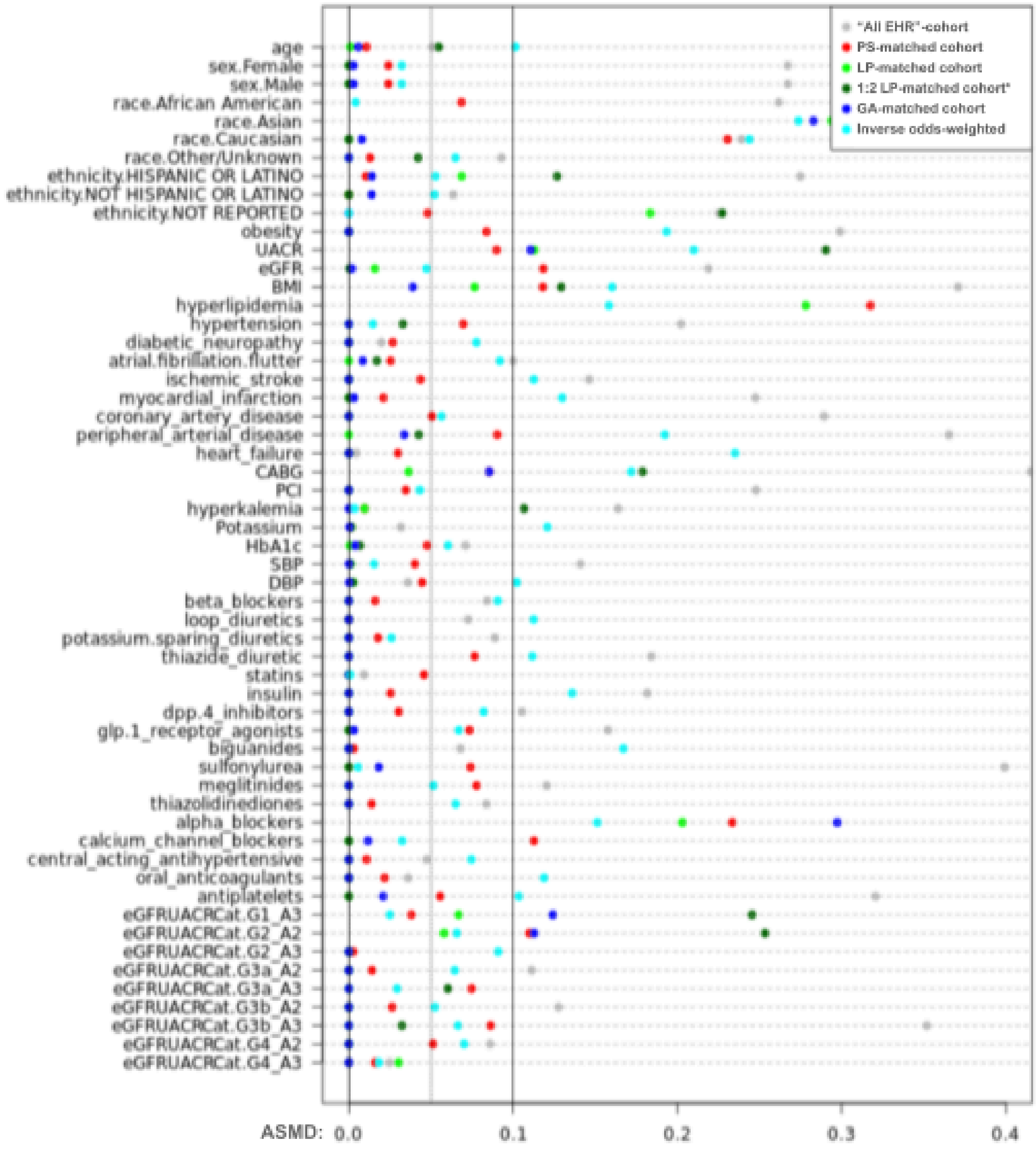

	Min	25% quantile	Mean	Median	75% quantile	Max	SD
**PS-matched cohort**	0.000	0.017	0.060	0.042	0.077	0.318	0.068
**LP-matched cohort**	0.000	0.000	0.033	0.000	0.004	0.409	0.083
**1:2 LP-matched cohort***	0.000	0.000	0.068	0.000	0.046	0.635	0.140
**GA-matched cohort**	0.000	0.000	0.037	0.000	0.012	0.460	0.096
**Inverse odds-weighted**	0.000	0.040	0.088	0.069	0.119	0.274	0.066

The chart shows SMDs for the utilized variables, matched with propensity score (PS), linear programming (LP), linear programming with ratio 1:2 RCT vs ECA patients, generic algorithm (GA) and the inverse odds-weighting approach. Chart area zoomed into region from 0-0.4.

Supplementary Figure S.3. Empirical cumulative distribution for continuous variables used for matching.

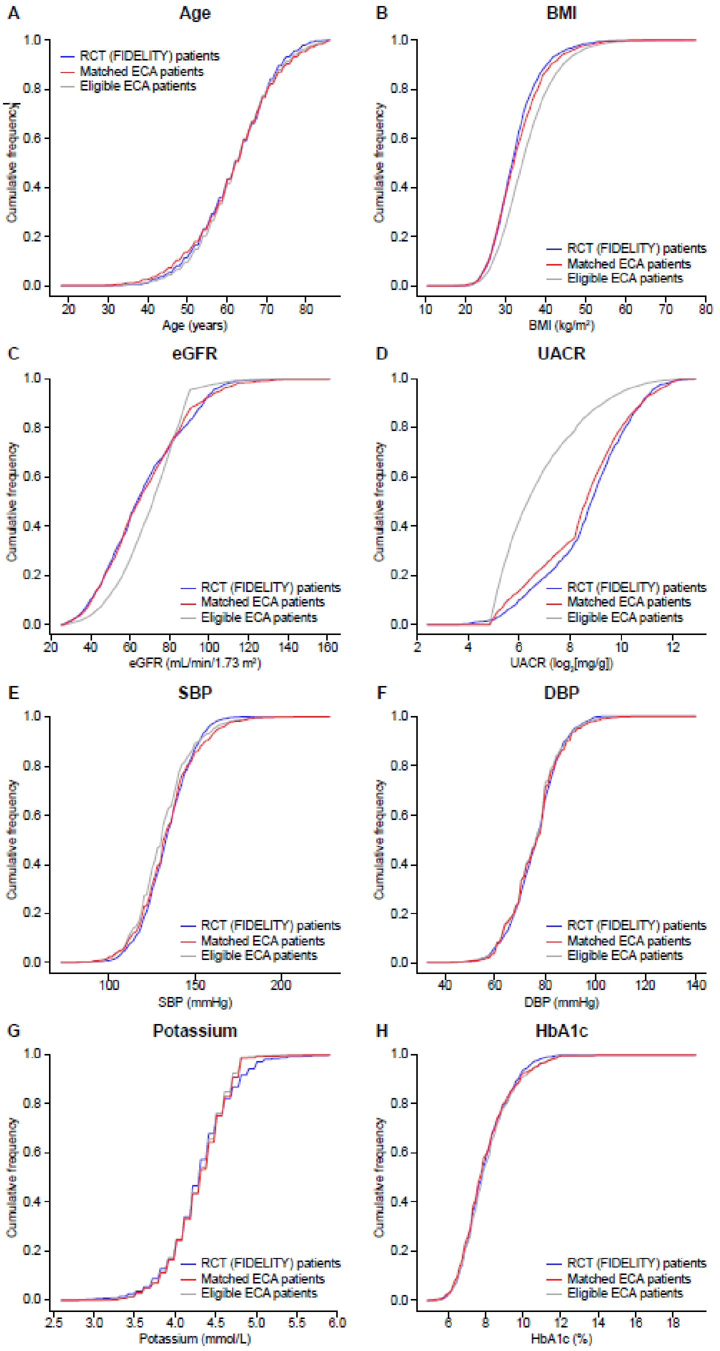

Empirical cumulative distributions for numerical variables used for matching for linear programming.

Abbreviations: BMI, body mass index; DBP, diastolic blood pressure; ECA, external control arm; eGFR, estimated glomerular filtration rate; HbA1c, glycated hemoglobin; RCT, randomized controlled trial; SBP, systolic blood pressure; UACR, urine albumin-to-creatinine ratio.

Supplementary Figure S.4. Variable frequency for ECA cohorts.

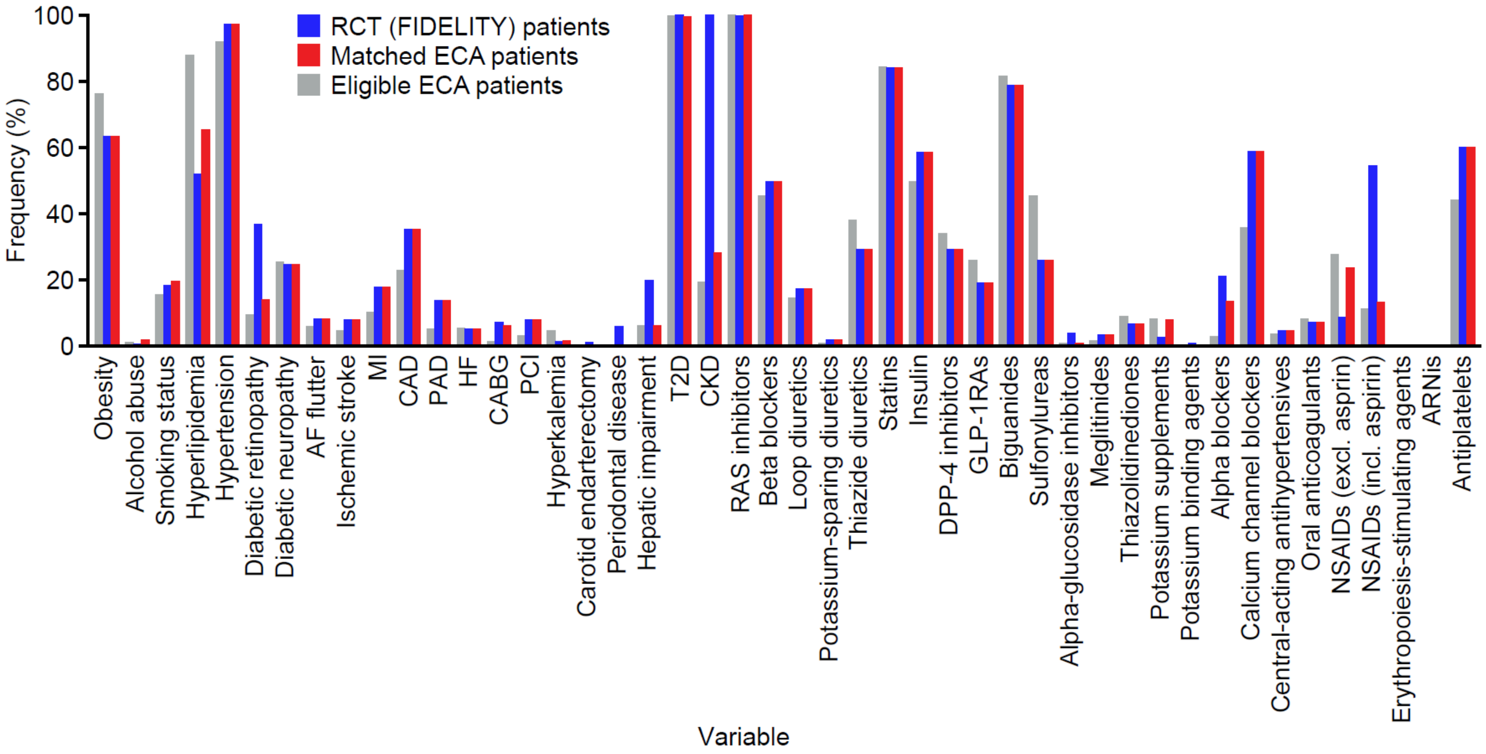

Frequencies of binary variables for ECA cohorts created by linear programming matching.

Abbreviations: ARNI; angiotensin receptor-neprilysin inhibitor; CABG, coronary artery bypass graft; CAD, coronary artery disease; CKD, chronic kidney disease; DPP-4 dipeptidyl peptidase-4; ECA, external control arm; GLP-1RA, glucagon-like peptide-1 receptor agonist; HF, heart failure; MI, myocardial infarction; NSAID, nonsteroidal anti-inflammatory drug; PAD, peripheral artery disease; PCI, percutaneous coronary intervention; RAS, renin–angiotensin system; RCT, randomized controlled trial; T2D, type 2 diabetes.

STROBE Statement

Checklist of items that should be included in reports of observational studies

	Item No	Recommendation	Page No
**Title and abstract**	1	(*a*) Indicate the study's design with a commonly used term in the title or the abstract	1
		(*b*) Provide in the abstract an informative and balanced summary of what was done and what was found	2
**Introduction**			
Background/rationale	2	Explain the scientific background and rationale for the investigation being reported	3–4
Objectives	3	State specific objectives, including any prespecified hypotheses	4
**Methods**			
Study design	4	Present key elements of study design early in the paper	4–7
Setting	5	Describe the setting, locations, and relevant dates, including periods of recruitment, exposure, follow-up, and data collection	4–7
Participants	6	(*a*) *Cohort study*—Give the eligibility criteria, and the sources and methods of selection of participants. Describe methods of follow-up<br>*Case-control study*—Give the eligibility criteria, and the sources and methods of case ascertainment and control selection. Give the rationale for the choice of cases and controls<br>*Cross-sectional study*—Give the eligibility criteria, and the sources and methods of selection of participants	4–7, Supplementary Table S.1.
		(*b*) *Cohort study*—For matched studies, give matching criteria and number of exposed and unexposed<br>*Case-control study*—For matched studies, give matching criteria and the number of controls per case	6,8, Supplementary Table S.2
Variables	7	Clearly define all outcomes, exposures, predictors, potential confounders, and effect modifiers. Give diagnostic criteria, if applicable	6–8, 11
Data sources/ measurement	8*	For each variable of interest, give sources of data and details of methods of assessment (measurement). Describe comparability of assessment methods if there is more than one group	6–8
Bias	9	Describe any efforts to address potential sources of bias	7–8, 11
Study size	10	Explain how the study size was arrived at	5–9
Quantitative variables	11	Explain how quantitative variables were handled in the analyses. If applicable, describe which groupings were chosen and why	7–8

Statistical methods	12	(*a*) Describe all statistical methods, including those used to control for confounding	7–8, 11–14
		(*b*) Describe any methods used to examine subgroups and interactions	7–8
		(*c*) Explain how missing data were addressed	7–8, 11–14
		(*d*) *Cohort study*—If applicable, explain how loss to follow-up was addressed<br>*Case-control study*—If applicable, explain how matching of cases and controls was addressed<br>*Cross-sectional study*—If applicable, describe analytical methods taking account of sampling strategy	NA
		(*e*) Describe any sensitivity analyses	NA

Results

Participants	13 *	(a) Report numbers of individuals at each stage of study—eg numbers potentially eligible, examined for eligibility, confirmed eligible, included in the study, completing follow-up, and analysed	8, Figure 1
		(b) Give reasons for non-participation at each stage	7–8
		(c) Consider use of a flow diagram	Figure 1
Descriptive data	14 *	(a) Give characteristics of study participants (eg demographic, clinical, social) and information on exposures and potential confounders	8, 24–25 (Table 1)
		(b) Indicate number of participants with missing data for each variable of interest	NA
		(c) *Cohort study*—Summarise follow-up time (eg, average and total amount)	24 (Table 1)
Outcome data	15 *	*Cohort study*—Report numbers of outcome events or summary measures over time	8–9
		Case-control study—Report numbers in each exposure category, or summary measures of exposure	NA
		Cross-sectional study—Report numbers of outcome events or summary measures	NA
Main results	16	(*a*) Give unadjusted estimates and, if applicable, confounder-adjusted estimates and their precision (eg, 95% confidence interval). Make clear which confounders were adjusted for and why they were included	8–9
		(*b*) Report category boundaries when continuous variables were categorized	8–9
		(*c*) If relevant, consider translating estimates of relative risk into absolute risk for a meaningful time period	NA
Other analyses	17	Report other analyses done—eg analyses of subgroups and interactions, and sensitivity analyses	8–9

Discussion

Key results	18	Summarise key results with reference to study objectives	9–11
Limitations	19	Discuss limitations of the study, taking into account sources of potential bias or imprecision. Discuss both direction and magnitude of any potential bias	10–14

Interpretation	20	Give a cautious overall interpretation of results considering objectives, limitations, multiplicity of analyses, results from similar studies, and other relevant evidence	9–14
Generalisability	21	Discuss the generalisability (external validity) of the study results	9–14
**Other information**			
Funding	22	Give the source of funding and the role of the funders for the present study and, if applicable, for the original study on which the present article is based	14

*Give information separately for cases and controls in case-control studies and, if applicable, for exposed and unexposed groups in cohort and cross-sectional studies.

Note: An Explanation and Elaboration article discusses each checklist item and gives methodological background and published examples of transparent reporting. The STROBE checklist is best used in conjunction with this article (freely available on the Web sites of PLoS Medicine at http://www.plosmedicine.org/, Annals of Internal Medicine at http://www.annals.org/, and Epidemiology at http://www.epidem.com/). Information on the STROBE Initiative is available at www.strobe-statement.org.
